# Divergent *in vitro/in vivo* responses to drug treatments of highly aggressive NIH-Ras cancer cells: a PET imaging and metabolomics-mass-spectrometry study

**DOI:** 10.18632/oncotarget.10470

**Published:** 2016-07-07

**Authors:** Daniela Gaglio, Silvia Valtorta, Marilena Ripamonti, Marcella Bonanomi, Chiara Damiani, Sergio Todde, Alfredo Simone Negri, Francesca Sanvito, Fabrizia Mastroianni, Antonella Di Campli, Gabriele Turacchio, Giuseppe Di Grigoli, Sara Belloli, Alberto Luini, Maria Carla Gilardi, Anna Maria Colangelo, Lilia Alberghina, Rosa Maria Moresco

**Affiliations:** ^1^ Institute of Molecular Bioimaging and Physiology, National Research Council (IBFM-CNR), Segrate, Italy; ^2^ SYSBIO.IT, Centre of Systems Biology, Milano, Italy; ^3^ Experimental Imaging Center, IRCCS San Raffaele Scientific Institute, Milan, Italy; ^4^ Department of Medicine and Surgery, University of Milano-Bicocca, Monza, Italy; ^5^ Tecnomed Foundation of University of Milano-Bicocca, Monza, Italy; ^6^ Department of Agricultural and Environmental Sciences - Production, Landscape, Agroenergy University of Milan, Milan, Italy; ^7^ Mouse Histopathology Unit, Department of Pathology, IRCCS San Raffaele Scientific Institute, Milan, Italy; ^8^ Institute of Protein Biochemistry, National Research Council, Naples, Italy; ^9^ Department of Biotechnology and Biosciences, University of Milano-Bicocca, Milan, Italy

**Keywords:** tumor, metabolic rewiring, PET-imaging, metabolomics-mass-spectrometry, oncogenic-K-ras

## Abstract

Oncogenic *K-ras* is capable to control tumor growth and progression by rewiring cancer metabolism. *In vitro* NIH-Ras cells convert glucose to lactate and use glutamine to sustain anabolic processes, but their *in vivo* environmental adaptation and multiple metabolic pathways activation ability is poorly understood. Here, we show that NIH-Ras cancer cells and tumors are able to coordinate nutrient utilization to support aggressive cell proliferation and survival. Using PET imaging and metabolomics-mass spectrometry, we identified the activation of multiple metabolic pathways such as: glycolysis, autophagy recycling mechanism, glutamine and serine/glycine metabolism, both under physiological and under stress conditions. Finally, differential responses between *in vitro* and *in vivo* systems emphasize the advantageous and uncontrolled nature of the *in vivo* environment, which has a pivotal role in controlling the responses to therapy.

## INTRODUCTION

Decades of studies and advances in high throughput techniques have identified the complex connection between oncogenic signaling pathways, metabolic reprogramming and increased nutrient uptake in cancer cells. Recently, Hanahan and Weinberg have added a state of “deregulated cellular energetics” to the original hallmarks of cancer, stressing the fact that cellular metabolism is substantially altered during oncogenesis and tumor progression [1]. The enhanced glycolytic activity and impaired oxidative phosphorylation (Warburg effect) characterizing tumors have been known for several years and represent the biological basis of the wide diffusion of [^18^F]FDG PET in diagnostic oncology [2]. More recently, it has been demonstrated that cancer cells can employ a large number of strategies to regulate cell homeostasis, which depend on different genetic or environmental factors (germline or somatic mutations, tissue vascularization, ongoing treatments, nutrients availability, immune system activity) [3–7]. In fact, activation of never-ending metabolic rewiring strategies tunes cancer cells to the continuous changes in environmental conditions, as it occurs in living organisms, to sustain enhanced growth and survival [8, 9]. Specifically, the metabolic rewiring shown by cancer cells involves not only the Warburg effect with enhanced glycolysis and high glutamine consumption, but also elevated rates of lipid biosynthesis, maintenance of redox homeostasis and limited levels of autophagy in the first steps of oncogenesis [10–12]. Recent evidence has shown that cancer cells can engage glutamine metabolism enzymes, such as PEP-carboxykinase (PCK2) [13] or pyruvate carboxylase (PC) [14], to develop metabolic adaptation and promote enhanced cell growth when even glucose is limited. In addition, they can activate autophagy when glutamine is inhibited [15]. Furthermore, metabolites, such as ATP, acetyl-CoA (AcCoA), reactive oxygen species (ROS) and enzymes with key role in metabolic regulation may act on different signaling pathways controlling tumor growth [9, 16–19]. However, the resulting effect of several cancer metabolic strategies is an abnormal NAD(P)H/NAD(P) balance ratio, which appears to promote glutamine utilization by reductive carboxylation to sustain amino acids and lipids synthesis and for ROS quenching [4, 10, 11, 20, 21].

Several findings indicated that mutations in oncogenes like *ras* and *myc* control tumor growth and spread by acting also on cell metabolism [22–25]. We have recently demonstrated that NIH3T3 fibroblasts harboring an oncogenic *K-ras* gene (NIH-Ras-G12V; transformed) [26] are able to decouple glucose and glutamine metabolism to support cancer cell growth [27]. In particular, using this simple tumor cell model, we observed that *K-ras* mutation induced *per se* a metabolic reprogramming that involved a decoupling of glucose and glutamine metabolism and enabled the efficient utilization of both carbon and nitrogen from glutamine for biosynthetic processes. In addition, it was observed that defects in autophagy were able to compromise survival of cancer cells in nutrient starvation and block tumor growth in allograft models [28, 29]. Moreover, it was demonstrated that activating *ras* mutations or Ras-effector pathways induced autophagy, which is essential for starvation, survival and tumor growth [29]. Given these *in vitro* results, oncogenic *K-ras* could drive metabolic reprogramming to develop environmental adaptation and/or resistance to drug treatments especially *in vivo* where metabolic conditions are more advantageous. In fact, experiments on cell lines cannot recapitulate the wide heterogeneity of tumors that occurs in *in vivo* conditions [30]. Therefore, targeting cancer metabolism through both *in vitro* and *in vivo* studies represent an attractive investigation strategy, particularly for *ras*-driven tumors that represent a strong therapeutic challenge.

Here, we used a well-characterized cellular model to investigate the role of simultaneous multiple pathways activation in NIH-Ras cells both *in vitro* and *in vivo* xenografts nude mice model. In addition, we examined metabolic changes in response to metabolic target drug treatments.

To address these issues, we took advantage of combined metabolomics (gas-chromatography-mass spectrometry - GC/MS) and *in vivo* PET imaging ([^18^F]FDG and [^18^F]FLT) approaches, which had the advantages of: a) detailing, at molecular level and within the context of environmental conditions, genetic regulation, altered kinetic activity of enzymes and changes in metabolic reactions [31, 32] and b) imaging macro-parameters (glycolysis and cell proliferation), which represent the end-stage of simultaneous activation or inhibition of different metabolic processes and signaling.

This strategy allowed us to verify whether information derived from PET studies had a sufficient diagnostic power in describing lesion phenotypes under different environmental conditions (in our case, perturbation of glutamine metabolism) and to validate targets for a theranostic approach of *K-ras* mutated cancer.

Our data indicated that gas-chromatography-mass spectrometry and PET imaging techniques allowed a complementary characterization of the NIH-Ras tumor model. Specifically, *in vitro* NIH-Ras cells were glutamine- and autophagy-dependent and inhibition of both pathways led to cell death. *In vivo*, NIH-Ras tumors maintained an aggressive phenotype, with high cell proliferation and glucose uptake. Following administration of inhibitors of autophagy and/or of glutaminase, tumors engaged in multiple metabolic processes to overcome the inhibition.

## RESULTS

### *In vitro* NIH-Ras cancer fibroblasts activate multiple pathways to sustain enhanced proliferation

It is well known that autophagy recycles intracellular components into metabolic pathways to sustain demand for growth and proliferation [33]. In accordance with published data [24, 27–29], oncogenic *K-ras* could cause multiple pathways activation, such as basal autophagy combined with increased glutamine metabolism, in order to sustain environmental adaptation and aggressive cell proliferation in cancer cells *in vivo*.

**Figure 1:**
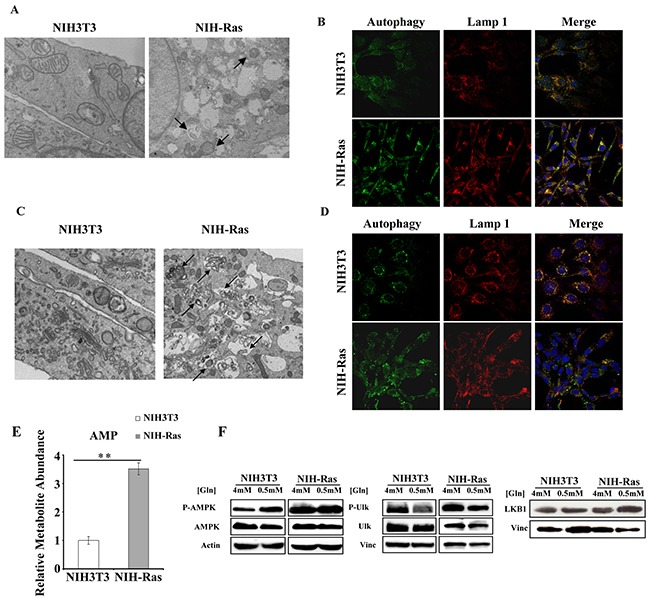
NIH-Ras cancer cells show autophagy activation to sustain enhanced cell proliferation **A, C.** Morphological analysis of NIH3T3 and NIH-Ras transformed cells by electron microscopy after 54h under normal growth conditions (4mM Gln) (A) or nutrient deprivation (0.5mM Gln) (C); arrows indicate the mitochondria within lysosomes. **B, D.** Analysis of autophagy and lysosomes organelles in NIH3T3 and NIH-Ras mouse fibroblasts cells under normal growth conditions (4mM Gln) (B) or nutrient deprivation (0.5mM Gln; D) by fluorescence microscopy using Cyto-ID® Autophagy detection kit (green color) and immunostaining for Lamp1 (red color); Hoechst was used for nuclei staining (Blue color). **E.** Relative AMP abundance in NIH3T3 and NIH-Ras mouse fibroblasts grown under conditions of glutamine deprivation for 54 h. AMP was measured by GC/MS and error bars indicate SD (n=3). **F.** Protein expression analysis of NIH3T3 and NIH-Ras cell lines grown in media containing 4mM glutamine (optimal growth condition) or 0.5mM glutamine (nutrient starvation). Cells were collected 54h after treatment and 30μg proteins from the total cellular extracts were processed by SDS-PAGE followed by Western blotting with the indicated antibodies.

To test the activation of basal autophagy mechanisms in our cellular model, we performed bioimaging analyses (Figure 1). Specifically, we found that NIH-Ras under normal nutrient conditions (4mM glutamine -Gln- and 25mM glucose) showed both an increased number of mitochondria within lysosomes (Figure 1A) and a higher number of lysosomes, as indicated by the lysosomal marker Lamp1 as compared to NIH3T3 (Figure 1B, upper panels) consistent with an increase of autophagy mechanism (Figure 1B, lower panels). These results were confirmed by measurements of fluorescence intensity (98.1 RSU measured in NIH-Ras, as compared to 64.18 RSU in NIH3T3) shown in Supplementary Figure S1A.

To further assess the role of basal autophagy in tumor growth, we performed experiments in response to glutamine starvation (0.5mM Gln) [24, 27]. Nutritional stress for 54h induced a remarkable increase of autophagic processes only in transformed cells, as revealed by the enhanced number of mitochondria within lysosomes (Figure 1C) and by the lysosomial marker Lamp1 (Figure 1D). Autophagy activation correlated with a significant increase of relative AMP abundance (Figure 1E) and increased levels of AMPK, P-AMPK, ULK1, P-ULK1 and LKB1 proteins in NIH-Ras cells both in normal (4mM) and glutamine deprived conditions (0.5mM Gln), as compared to NIH3T3 cells (Figure 1F and Supplementary Figure S1B-C and S1D).

**Figure 2:**
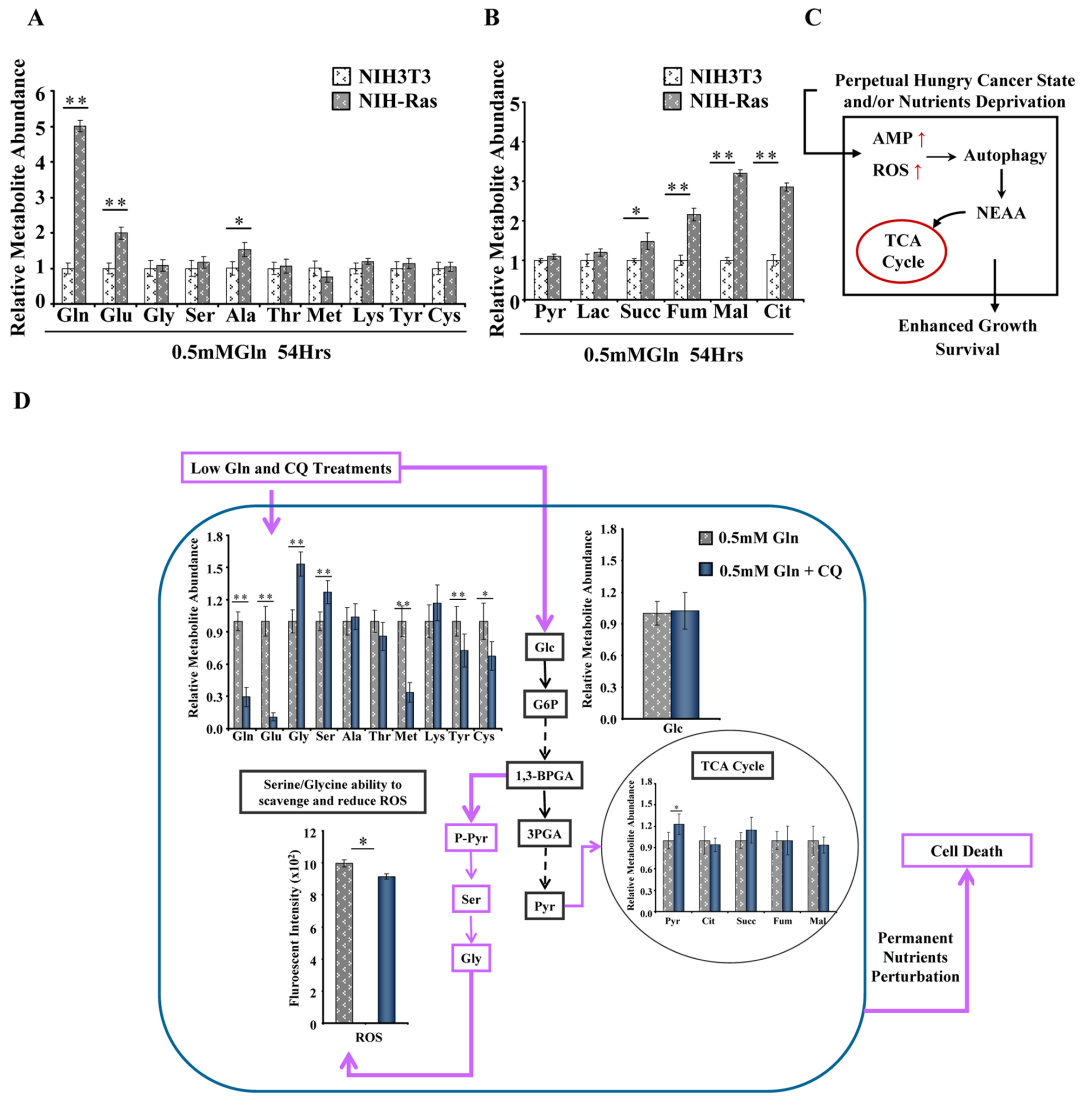
Basal autophagy recycle of metabolites and alternative pathways activation in NIH-Ras cancer cells **A** and **B.** Relative metabolites abundance, free amino acids (A) and TCA cycle metabolites (B), measured by GC/MS in NIH3T3 and NIH-Ras cells cultured in low glutamine (0.5mM Gln) for 54h. **C.** Schematic representation summarizing how cancer metabolic ability in sustaining permanent nutrient request takes advantage of autophagy mechanisms induced by high ROS and AMP levels to provide amino acids to the TCA cycle. **D.** Schematic representation of basal autophagy recycle of metabolites and alternative pathways activation. Relative metabolites abundance performed by GC/MS in NIH-Ras cells grown in 0.5mM Gln or 0.5mM Gln plus chloroquine (CQ, 50μM) for 54h. Error bars indicate SD (n=5). Intracellular ROS levels measured by DCFDA staining in NIH-Ras grown in medium containing 0.5mM Gln or 0.5mM Gln plus CQ for 54h. Error bars indicate SD (n=3).

In addition, the metabolic profiling of NIH-Ras grown under glutamine deprivation displayed increased levels of free amino acids, mainly glutamine (Figure 2A), and TCA cycle metabolites (Figure 2B), suggesting that replacement of metabolites at different levels could be provided via autophagy to ensure nutrient demand under stress and satisfy the permanent hungry state (Figure 2C) [29]. As expected, the metabolic profiling of NIH-Ras under metabolic perturbation with low glutamine plus chloroquine (CQ), an inhibitor of autophagosomal degradation [34, 35] showed a decreased relative abundance of glutamine, glutamate and other amino acids, as compared to low glutamine conditions (Figure 2D).

**Figure 3:**
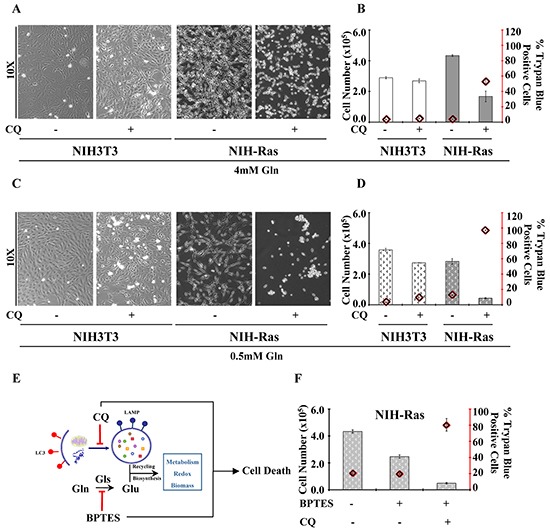
Aggressive proliferative K-Ras transformed fibroblasts show glutamine-dependent metabolic rewiring **A.** Morphological analysis of NIH3T3 and NIH-Ras transformed cells cultured in normal growth conditions CTRL or treatment with CQ (50μM) for 24h. Cells were observed at phase-contrast microscopy. **B.** Percentage of NIH3T3 and NIH-Ras trypan blue positive cells cultured under normal growth conditions CTRL or after treatment with CQ (50μM) for 24h. **C.** Morphological analysis of NIH3T3 and NIH-Ras transformed cells cultured in 0.5mM Gln CTRL in the absence or presence of CQ (50μM) for 24h. **D.** Percentage of normal and transformed trypan blue positive cells cultured in 0.5mM Gln CTRL in the absence or presence of CQ (50μM) for 24h. **E.** Schematic representation of autophagy and glutamine metabolism sustaining cancer growth and their inhibition by BPTES and CQ treatments. **F.** Proliferation curve of NIH-Ras mouse fibroblasts treated with BPTES, or CQ or their combination. Cells were plated at 3000/cm^2^. After 18h cells were treated with BPTES (6μM) or CQ (50μM) or BPTES+CQ and counted after 54 h. Error bars indicate SD (n=3).

On the other hand, we also observed significant increased levels of serine (Ser) and glycine (Gly) (Figure 2D), but we did not find significant changes in intracellular relative abundance of glucose and TCA cycle metabolites in NIH-Ras grown in low glutamine or low glutamine plus CQ (Figure 2D). Taken together, these results suggest that the strong metabolic stress due to low glutamine plus CQ (Figure 2D) or CQ treatment alone (Supplementary Figure S2A and S2B) could lead NIH-Ras cells to use glucose (Figures 2D and S2A) in alternative pathways, such as serine/glycine pathway (Figures 2D and S2B) [36], in the attempt to decrease ROS levels (Figure 2D and S2C) and trying (unsuccessfully) to survive (Figure 2D). In fact, as shown in Figure 3, CQ treatment induced cell death in NIH-Ras cells grown both in normal growth medium (4mM Gln) (Figure 3A–3B) (trypan blue positive cells 53%, red diamond) and following glutamine starvation for 54h (Figure 3C–3D) (trypan blue positive cells 97.2%, red diamond).

However, although autophagy seemed to play an important role in sustaining cancer growth and survival during short glutamine deprivation (54h), we observed that this mechanism was not able to maintain survival when the glutamine deprivation was prolonged for 144h (Supplementary Figure S2D, left and right panels). Specifically, NIH-Ras transformed cells showed a larger decreased cell number when grown under prolonged nutritional stress (Supplementary Figure S2D, right panel), as compared to cells grown in multi-strata in normal growth conditions (Supplementary Figure S2D, left panel). On the other hand, we did not observe differences in NIH3T3 proliferation (Supplementary Figure S2D, left panel), whose decreased cell number at 144h is known to be due to confluence and physiological contact-dependent death program (anoikis) [37]. Taken together our results identified the key role of autophagy recycling mechanisms and glutamine metabolism in sustaining anabolic processes, redox balance, cancer survival and aggressive cancer proliferation (Figure 3E). To confirm the role of low glutamine on cell proliferation, we assessed the effect of different concentrations of BPTES (bis-2-(5-phenylacetamido-1,2,4-thiadiazol-2-yl)ethyl sulfide), an inhibitor of glutaminase activity (Supplementary Figure S2E). Consistent with previous results, we found that BPTES (4, 6 or 8μM) reduced NIH-Ras cells proliferation, as compared to CTRL (Supplementary Figure S2E), without changing cell proliferation in NIH3T3. Finally, to obtain a synergic effect we performed a simultaneous perturbation of both pathways (autophagy and glutamine) by using CQ and BPTES together. NIH-Ras cells treated with CQ and BPTES showed a dramatic reduction of proliferation (Figure 3F) with 81% of trypan blue positive cells (Figure 3F, red diamond), as compared to cells grown.

All together, these data suggest that the combination of BPTES and CQ could be an interesting intervention strategy to be tested *in vivo*.

### PET imaging and metabolomics-mass-spectrometry revealed a time-dependent metabolic shift in NIH-Ras model

**Figure 4:**
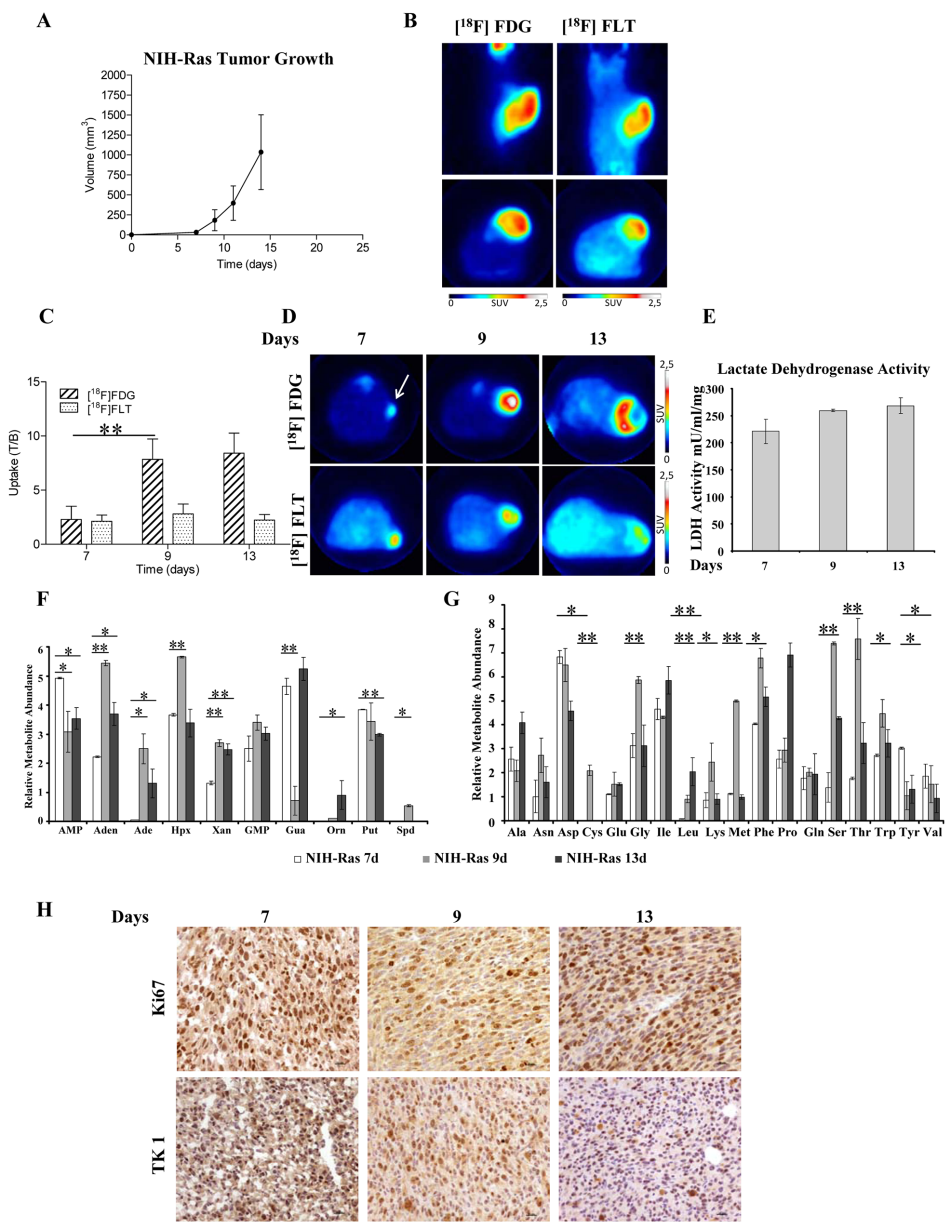
PET imaging and metabolomics technique identify fast growth and highly aggressive phenotype of NIH-Ras tumor xenografts *in vivo* **A.** NIH-Ras tumor xenografts growth curve was established by caliper measurement after s.c. injection of NIH-Ras cancer cell fibroblasts. **B.** Representative [^18^F]FDG and [^18^F]FLT PET coronal and transaxial images of NIH-Ras tumors performed at 10 days after cells injection. Color scale is expressed as SUV. **C.** [^18^F]FDG and [^18^F]FLT uptake expressed as T/B ratio at different time points in NIH-Ras tumors. From day 7 to day 9 [^18^F]FDG uptake significantly increased (p<0.001). Error bars indicate SD (n=5-8 mice per PET study). **D.** Representative [^18^F]FDG and [^18^F]FLT transaxial images of NIH-Ras tumors at different time points from cell injection. Color scale is expressed as SUV and the white arrow indicates the tumor. **E.** LDH enzyme activity of NIH-Ras tumor xenografts from 7 to 13 days. LDH enzyme activity was measured by enzymatic assay and normalized to protein content. Error bars indicate SD (n=3). **F** and **G.** Relative metabolites abundance involved in nucleotides (F) and amino acids (G) metabolism of NIH-Ras tumor xenografts from 7 to 13 days measured by GC/MS. **H.** Ki67 and TK1 IHC staining of NIH-Ras tumors collected from mice at 7, 9 and 13 days after cells injection.

To determine whether the nutrient utilization rewiring observed *in vitro* reflected the same metabolic dependencies *in vivo*, we evaluated NIH-Ras cells in a mouse model of xenograft. In particular, we monitored tumor growth (using caliper), glucose metabolism ([^18^F]FDG-PET) and cell proliferation ([^18^F]FLT-PET). First, we characterized *in vivo* tumor growth using caliper and PET with [^18^F]FDG and [^18^F]FLT starting when tumors were palpable. Tumor lesions rapidly grew and 100% of animals showed palpable tumors 7 days after cells injection (Figure 4A). Ten days after cells injection, animals performed PET scan and images showed the presence of glucose-avid and highly proliferative cancer lesions (Figure 4B). Within 15 days tumors reached volumes of 1090 ± 456 mm^3^ (Figure 4A) and animals had to be sacrificed to avoid suffering. The rapid growth of NIH-Ras cells observed, probably due to a more advantageous *in vivo* environment as compared to *in vitro* conditions, highlighted NIH-Ras immortalized cancer cells as a highly aggressive *in vivo* model.

The time-course of lesions progression performed in additional groups of animals confirmed the hyper-proliferative phenotype that was maximum at 7 days after tumor implantation and remained stable thereafter. On the contrary, an abrupt increase of [^18^F]FDG was observed between 7 and 9 days, which remained stable until the 13^th^ day (Figure 4C-4D). As previously observed, tumors rapidly grew in parallel with increased [^18^F]FDG or [^18^F]FLT volumes (Supplementary Figure S3A and S3B); moreover, measurements of lactate dehydrogenase (LDH) activity in tumors collected after mice sacrifice confirmed the presence of Warburg effect (Figure 4E). On the other hand, we also observed a low *in vivo* hexokinase (HK) activity (Supplementary Figure S3C), consistent with our NIH-Ras cancer cells transcriptomics published data [27], showing lower HK gene expression as compared to normal cells, suggesting ADP-glucokinase (glucose-phosphorylating enzyme) as an alternative enzymatic source [38] to support the high glycolytic metabolism and greater advantage in ATP production. Moreover, consistently with the rapid growth and [^18^F]FLT uptake, we found increased levels of metabolites involved in nucleotide synthesis (Figures 4F and S3D) between day 7 and day 9. Specifically, we observed increased relative abundance of adenosine (Aden), adenine (Ade), hypoxanthine (Hpx), xanthine (Xan), GMP and spermidine (Spd) (Figure 4F). Furthermore, we found significant changes in the relative abundance of various amino acids (Figure 4G). Notably, the majority of essential and non-essential amino acids were more abundant at day 9, as compared to day 7 and day 13 (Figure 4G).

To investigate morphological features and to correlate *in vivo* data with proliferation markers, *post mortem* H&E analysis and IHC confirmed the high aggressiveness of NIH-Ras tumors. Samples displayed high mitotic activity regardless of the time points, with only small necrotic areas present mainly at 13 days (Figure 4H). IHC staining revealed tumors with homogeneous and high positivity for both Ki67 and TK-1 antibodies that slowly increased over time (Figure 4H), thus confirming [^18^F]FLT PET data.

Taken together, these results indicated a metabolic shift between 7 to 9 days (glucose uptake and amino-acid levels), which could be a potential therapeutic window to maximize the effects of treatments that target cell metabolism.

### Highly aggressive NIH-Ras tumor response to metabolic stress conditions detected by PET imaging and metabolomic-mass-spectrometry

**Figure 5:**
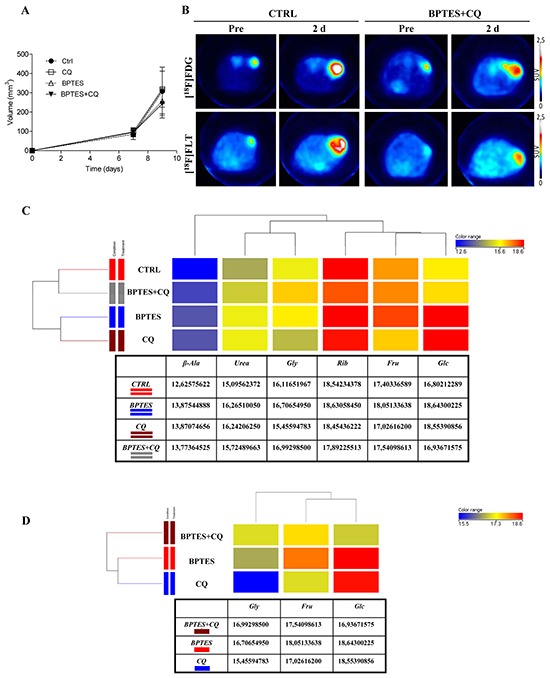
BPTES glutaminase inhibitor and CQ autophagy inhibitor do not show anti-proliferative activity in highly aggressive NIH-Ras tumors **A.** Evaluation of tumor size after treatment. Changes in tumor size were measured by caliper in mice treated with a 2-day scheduled treatment. Mice received daily vehicle (CTRL), or CQ (50mg/kg), or BPTES (10 mg/kg), or BPTES plus CQ (Combination). **B.** Representative transaxial [^18^F]FDG and [^18^F]FLT PET images of CTRL and combined treatment mice performed before and after drugs administration. Color scale is expressed as SUV value. **C.** Comparison of samples by one way ANOVA statistical analysis (CTRL *versus* single treatments). **D.** Comparison of samples by one way ANOVA statistical analysis (BPTES and CQ combined treatment *versus* single treatments). ANOVA statistical analysis was performed using Mass Profiler Professional (MPP) software. The dendrogram was produced by applying a hierarchical clustering algorithm. The color range legends was automatically generated by MPP, considering the minimum and maximum values of most compounds identified to highlight the best differences between samples through the most suitable color scale. Quantitative values of relative metabolites abundance are shown in the tables.

To evaluate *in vivo* metabolic changes in drug treatment response and to validate PET imaging and metabolomic-mass-spectrometry as complementary diagnostic tools, mice with NIH-Ras tumors were treated with BPTES and CQ, alone or in combination, using the therapeutic windows previously identified, i.e. at 7 days. In contrast to *in vitro* results, surprisingly, BPTES alone or in combination with CQ failed to arrest tumor growth (Figure 5A), glucose uptake ([^18^F]FDG-PET) and cell proliferation ([^18^F]FLT-PET) (Figures 5B and S4A-B). In addition, *post mortem* data revealed no significant difference in glutamate levels between CTRL and BPTES treated mice (Supplementary Figure S4C) and showed the collateral effect of inducing autophagy (Supplementary Figure S4D) [15].

Consistent with PET findings, metabolomics analysis did not show significant changes either in metabolite composition or in metabolite abundance across all treatments (Supplementary Figure S5A). Hierarchical clustering of samples was performed on normalized intensities of 85 significant metabolites (Supplementary Figure S5A) followed by pathways enrichment of nucleotide metabolism (Supplementary Figure S5B, left panel), glycolysis and TCA cycle (Supplementary Figure S5B, right panel). The resulting dendrogram, in which the length of the vertical lines connecting two groups of samples is proportional to the distance between them, confirmed the close similarity among groups. Although all groups are similar to each other, CTRL and combined treatment clustered together (Supplementary Figure S5A), whereas BPTES and CQ pertained to a separate cluster. In addition, 6 metabolites (β-alanine, urea, glycine, ribose, fructose and glucose) out of 85 were found to be significantly different across samples by using one way ANOVA (Figure 5C). Specifically, we found increased relative abundance of glucose (Glc) and Urea in all treatments, as compared to CTRL (Figure 5C). In addition, we observed significant increased relative abundance of fructose (Fru) and glycine (Gly) in BPTES and BPTES plus CQ treatments, respectively, as compared to CTRL. After comparison of the four groups by ANOVA, t-test was applied in order to find out which metabolites were more significantly expressed in single treatment groups *versus* CTRL (Supplementary Figure S5C and S5D). In particular, we found significant increased relative abundance of glucose, urea and β-alanine (β-Ala) metabolites in BPTES and CQ treatments *versus* CTRL (Supplementary Figure S5C and S5D, respectively), suggesting a common metabolic response of NIH-Ras tumors to single drug treatments. At the same time, increased relative abundance of metabolites, such as creatinine (Cr), aspartate (Asp) and fructose (Fru) in BPTES treatment, and increased relative abundance of uric acid and hypoxanthine (Hyp) in CQ were found statistically significant in the two groups, as compared to CTRL (Supplementary Figure S5C and S5D, respectively).

To further highlight differences between treatments, one way Anova analysis (Figure 5D) confirmed BPTES and CQ clustering, as indicated by increased relative abundance of glucose in both single treatments *versus* combined treatments, and increased relative abundance of fructose in BPTES alone *versus* CQ or BPTES and CQ combined treatment (Figure 5D). On the other hand, we found increased relative abundance of glycine in the combined group, as compared to single treatments (Figure 5D). Taken together, these data emphasize that multiple metabolic pathway activation, due to advantageous environmental conditions, could cause the reduced effect of the drugs, at the same time sustaining aggressive cell proliferation of NIH-Ras tumors observed *in vivo* experiments [39].

## DISCUSSION

Metabolic rewiring following increased nutrients uptake sustains uncontrolled proliferation [6]. In particular, both oncogenic *ras* and *myc* stimulate glucose uptake and regulate glutamine metabolism, specifically directing glutamine carbons into pathways able to support biosynthesis, redox homeostasis, cell survival and enhanced growth [27, 40, 41]. A recent work has highlighted the importance of metabolic rewiring in both cultured cells and *in vivo* mice models [6]. It has been demonstrated that glucose deprivation, or growth in the restricted environment of the subcutaneous space in mice, renders transformed K-Ras cancer cells tolerant of low glucose conditions [42]. On the other hand, chronic exposure to low glucose still requires oxidative phosphorylation as a means to maintain growth [43]. Therefore, the complex metabolic requirements of cancer cells lead to greater metabolic flexibility able to activate multiple pathways using alternative substrates that may contribute to ATP synthesis by oxidative phosphorylation (OXPHOS) or intermediate synthesis necessary to sustain anabolic processes for enhanced growth and/or survival [27, 44]. Hence, tumor cells capable of using alternative substrates for metabolic rewiring modulation *in vivo* could develop never-ending strategies also under stress conditions.

In this study we used a well characterized *ras*-driven cancer cell model (NIH-Ras) both *in vitro* and *in vivo* to evaluate the role of Ras mutation in cancer lesions response to different environmental conditions using a metabolomics-mass-spectrometry and PET imaging combined approach.

**Figure 6:**
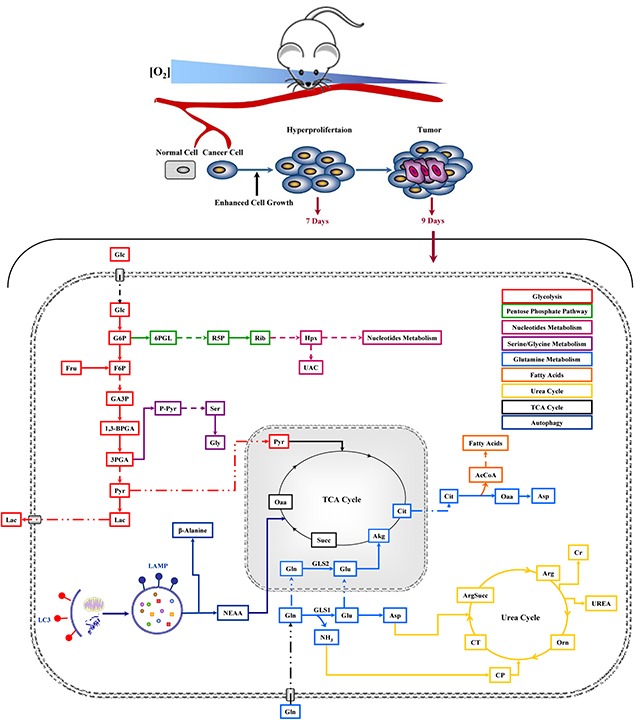
Schematic representation of cancer metabolic pathways identified in *in vivo* NIH-Ras tumor xenografts and the effect of metabolic interfering drugs Metabolic Profiling was performed 9 days after cell injection and after 48h of drug treatments.

Our results confirmed the glutamine addiction and showed an activation of basal-autophagy in the first steps of cancer cell growth (Figure 1). Under low glutamine condition, we observed an interesting increase of amino acids and TCA cycle metabolites, in particular glutamine, suggesting that autophagy replenishes TCA cycle intermediates to sustain survival (Figure 2) [29]. In agreement with this observation, blocking autophagy by CQ caused a massive cell death, which was further increased by the combined administration of the glutaminase inhibitor (BPTES) (Figure 3). Taken together, these results confirmed that in vitro NIH-Ras cancer cells took advantage not only of enhanced glycolysis, as depicted in the scheme in Figure 6 (red arrows), but also of glutamine metabolism (Figure 6, light-blue arrows) and autophagy recycling (Figure 6, blue arrows) to sustain cellular hyper-proliferation.

Based on these *in vitro* results confirming that well established multiple pathway activation sustains enhanced growth in NIH-Ras cancer cells, *in vivo* studies were performed with the purpose to provide a native microenvironment that is metabolically advantageous.

*In vivo*, NIH-Ras cells gave rise to aggressive lesions characterized by high proliferation rate ([^18^F]FLT) and glucose demand ([^18^F]FDG), which were increased between 7 and 9 days (Figure 4A–4D). Interestingly, in the same time-frame we observed an increase of metabolites involved in nucleotides synthesis (Figure 4F) and augmented relative abundances of various essential and not essential amino acids (Figure 4G). This metabolic shift confirmed that *in vivo* tumors are able to activate multiple pathways to create a more advantageous environment for cancer growth. In addition, as previously observed *in vitro* [27], we found reduced activity of HK (Supplementary Figure S2C), suggestive of the activation ADP-glucokinase, a more efficient hexokinase able to support a higher glycolytic demand [38]. Finally, to evaluate modifications of *in vivo* metabolism under stress conditions, BPTES and CQ, alone or in combination, were administered to NIH-Ras tumor xenografts. In contrast to our *in vitro* results, both drugs did not produce notable effects in the rate of lesions growth and PET parameters, particularly when CQ was given alone (Figure 5).

As for BPTES, our results are in line with the observations in BRAF mutated cells displaying high sensitivity to BPTES *in vitro* but not *in vivo* [45]. Probably, differently from *myc* driven lesions, where BPTES was able to suppress tumor growth [42], activation of Ras pathway gives to cancer cells higher ability to sustain tumor growth during metabolic stress.

It is worthy of note that metabolomics analysis showed significant changes in glucose, urea and β-alanine metabolites when BPTES and CQ were given alone (Figure 5). These results confirmed the key role of glucose (Figure 6, red arrows) and suggest the activation of the urea cycle pathway (Figure 6, yellow arrows) involved in ammonia removal and β-alanine involved in metabolic rewiring, like it occurs following epithelial-mesenchymal transition (EMT) [39, 46]. On the other hand, the significant increase in glycine levels found under a combined drugs treatment suggests that NIH-Ras tumors could activate multiple alternative pathways, such as the serine-glycine pathway [36], able to sustain the aggressive cell proliferation of NIH-Ras tumors in metabolic stress conditions (Figure 6, lilac arrows) [47, 48]. In such an adaptive system the block of glutaminase activity, as performed by BPTES [49], which spares other glutamine sources, a biosynthesis-dependent process is clearly not sufficient [39].

In addition, *in vivo* glutamine need is ensured by glutamine producer organs through an inter-organ glutamine trafficking that is modified in cancer to increase glutamine availability. For instance, in a mice model of fibrosarcoma, tumor lesion induces muscle expression of glutamine synthetase and greatly increases the release of glutamine into the circulation [50].

This systemic metabolic rearrangement characterizing cancer might also explain the lack of CQ effect, together with a progressive loss of autophagy dependence of NIH-Ras cells during tumor progression, even when CQ was administered before the activation of the alternative metabolic pathways, as previously reported [51].

In conclusion, in this study we demonstrated the amazing adaptation ability of *ras* driven tumors to environmental modifications and metabolic stress using metabolic rewiring and alternative pathways. Differential responses between *in vitro* and *in vivo* systems have been shown to be particularly evident under *in vivo* conditions where nutrients supply is continuously available from different body reserves and where probably systemic anabolic processes are also adapted to sustain cancer progression.

These observations may explain why one of the few therapeutic options for *ras* driven tumors is represented by drugs able to block angiogenesis with a general reduction of nutrient supply. This suggests that general metabolic sensors, which are able to regulate the activation of alternative pathways, should be considered. Finally, we showed that the combined use of PET imaging and metabolomics-mass spectrometry can detail at the molecular level the specific metabolic signature of tumors. This information might be relevant for validating the potential diagnostic power of metabolomics for clinical use in conjunction with the imaging parameters (in this case glycolysis and cell proliferation), which represent the end stage of the simultaneous activation or inhibition of different metabolic processes and signaling.

## MATERIALS AND METHODS

### Cell culture

Mouse embryonic fibroblast NIH3T3 cells (CRL-1658; American Type Culture Collection), K-Ras transformed NIH3T3-derived cell line 226.4.1 [52] were routinely grown in Dulbecco's modified Eagle's medium containing 4mM L-glutamine, 100U/ml penicillin and 100mg/ml streptomycin (normal growth medium) supplemented with 10% newborn calf serum at 37°C in a humidified atmosphere of 5% CO2. Cells were passed using trypsin-EDTA and maintained in culture for 54h or 144h before manipulation. All reagents for media were purchased from Life Technologies Invitrogen (Carlsbad, CA, USA).

### Cell proliferation analysis and cell treatments

Cells were plated at the density of 3000 cells/cm^2^ in complete growth medium. After 18h, cells were washed twice with phosphate-buffered saline (PBS) and incubated in a media with different glutamine concentrations (4mM or 0.5mM Gln).

For specific treatments, cells were treated with 50μM Chloroquine (CQ), or 4-6-8μM BPTES (Sigma-Aldrich Inc.). To measure cell proliferation, harvested cells were counted by Burker chamber and live/dead count was performed using trypan blue exclusion.

### Animal model

To generate the mouse model, NIH-Ras cells (2.5 × 10^5^) were injected subcutaneously (s.c) on the right flanks of 8-week old nu/nu female mice (Harlan Laboratories). Animals were kept under specific pathogen-free conditions, handled and maintained according to San Raffaele Institutional Animal Care and Use Committee (IACUC) ethical regulations, which previously approved the experiments described in this study. After cells injection, mice were monitored twice a week for body weight and tumor volume was measured using a digital caliper and calculated following the formula: tumor volume = (long side*(short side)^2)/2). At the end of studies, mice were sacrificed by CO_2_ asphyxiation and tumors collected for post mortem analysis.

### Study design characterization

Different groups of animals were evaluated using PET and [^18^F]FDG or [^18^F]FLT at 7, 9 and 13 days after cell injection (n=5-8 per time point and per radiotracer). At the end of PET study, animals were sacrificed and tumors collected for post mortem metabolomics and immuno-histochemistry analyses.

### Metabolic perturbation by pharmacological therapy

In vivo studies were performed using BPTES (Bis-2-[5-phenylacetamido-1,2,4-thiadiazol-2-yl]ethyl sulphide, intraperitoneal, i.p. 10mg/kg in 2% DMSO, Sigma-Aldrich®), an inhibitor of glutaminase enzyme and chloroquine (CQ, i.p. 50 mg/kg in PBS, Sigma-Aldrich), an inhibitor of autophagosomal degradation, alone or in combination. Mice were divided into 4 groups (n=3-5) of treatment (Vehicle, BPTES, CQ and Comb) and treated for 2 consecutive days starting 7 days after cell injection. Control animals received i.p. vehicle. Animals performed [^18^F]FDG and [^18^F]FLT PET before and after therapy.

### PET studies

PET images were obtained using a YAP-(S)-PET II small animal scanner (ISE s.r.l., Pisa, Italy). For [^18^F]FLT studies, 4.2±0.3 MBq were injected in the tail vein (i.v.). [^18^F]FLT production, acquisition and images quantification were performed as previously described [31]. [^18^F]FDG was prepared for clinical use (European Pharmacopeia VIII Edition) and i.v. injected (4.3±0.2 MBq) in animals in fasting conditions. The same procedure of acquisition and image analysis used for [^18^F]FLT was applied to [^18^F]FDG scan. Radioactivity concentrations were expressed as Standardized Uptake Value (SUVmax = (max radioactivity measured in the ROI/injected radioactivity)*animal weight) and tumor-to-background radioactivity concentration ratios (T/B).

### Post mortem analyses

For immunohistochemistry (IHC) studies, after sacrifice, cancer lesions were removed, dissected, fixed in 4% PFA, cryopreserved in liquid N_2_ cooled isopentane and embedded in OCT. Serial 6μm thick sections were treated with 0.3% H_2_O_2_ to quench endogenous peroxidase activity. Cell proliferation was evaluated using Ki67 and TK1 antibodies (Novus Biological) to correlate results with [^18^F]FLT data.

### Enzymatic assays

For enzymatic assay quantification of LDH Colorimetric Assay kit (BioVision), HK Colorimetric Assay kit (BioVision) and glutamate Colorimetric Assay kit (BioVision), 10mg of NIH Ras tumors were rapidly homogenized on ice in 0.1 ml of appropriate assay buffer and processed according to the manufacturer's protocols.

### ROS levels measurement

ROS levels were measured using the DCFDA Cellular Ros Detection Assay Kit from Abcam (Cambridge, UK). Cells were harvested and stained with 20μM dichloro-diyidro-fluoresceine-diacetate (DCFDA) for 30 min at 37°C. Thereafter, cells were washed and fluorescence was measured at excitation/emission wavelengths of 485nm/535nm respectively using Cary Eclipse Fluorescence Spectrophotometer.

### Metabolite profiling cell culture samples

Metabolites were extracted and analyzed by gas chromatography-mass spectrometry (GC/MS), as described previously [27]. Details are reported in Supplemental Experimental Procedures.

### Metabolite profiling in tissue samples

For tissue metabolite extraction, 0.1 ml ice-cold methanol was added to 10mg of tissue and incubated 5 min on ice. An equal volume of water was added and samples were sonicated 5 sec for 5 pulses at 70% power for three times. After a 30 min incubation at −80°C, samples were sonicated as described above. One volume of chloroform was added and samples were vortexed at 4°C for 30 min and then centrifuged at 12000g for 10 min. The aqueous phase was recovered and evaporated under airflow at 37°C [53].

Derivatization was performed using automated sample prep WorkBench instrument (Agilent Technologies) as detailed in the Supplemental Experimental Procedures.

Dried polar metabolites were dissolved in 60μl of 2% methoxyamine hydrochloride in pyridine (Pierce) and held at 40°C for 6h. After dissolution and reaction, 90μl of MSTFA (N-Methyl-N-(trimethylsilyl) trifluoroacetamid) were added and samples were incubated at 60°C for 1h.

GC/MS analysis was performed using 7200 accurate-mass Q-TOF GC/MS (Agilent Technologies) equipped with a 40-m DB-5MS capillary column operating under electron impact (EI) ionization at 70eV. Samples (2μl) were injected in a splitless mode at 250°C, using helium as the carrier gas at a flow rate of 1 ml/min. The GC oven temperature was held at 100°C for 2 min and increased to 325°C at 10°C/min.

GC/MS data processing was performed using Agilent Muss Hunter software and statistical analyses were performed using Mass Profile Professional (MPP) software [54]. Relative metabolites abundance was carried out after normalization to internal standard norvaline and cell number.

### Electron microscopy

Electron microscopy experiments were performed as described in Supplemental Experimental Procedures [55].

### Immunofluorescence microscopy

Protocols and antibodies are described in the Supplemental Experimental Procedures.

### Western blotting analysis

Protocols and antibodies are described in the Supplemental Experimental Procedures.

### Statistical analysis

Results are expressed as mean value ± SD. Experimental differences were tested for significance with the Student's *t*-test or, when possible, with the One Way ANOVA test. A p-value of 0.05 or less was considered statistically significant.

## SUPPLEMENTARY EXPERIMENTAL PROCEDURES


