# Supplementary material for: Divergent in vitro/in vivo responses to drug treatments of highly aggressive NIH-Ras cancer cells: a PET imaging and metabolomics-mass-spectrometry study
Source: Oncotarget. 2016 Jul 7;7(32):52017–31. doi: 10.18632/oncotarget.10470 (PMC5239532; doi:10.18632/oncotarget.10470)
Supplement: Supplementary file 1 [file oncotarget-07-52017-s001.pdf]

Divergent *in vitro/in vivo* responses to drug treatments of highly aggressive NIH-Ras cancer cells: a PET imaging and metabolomics-mass-spectrometry study

SUPPLEMENTARY EXPERIMENTAL PROCEDURES

List of abbreviation:

Glc Glucose
G6P Glucose-6-phosphate
F6P Fructose-6-phosphate
Fru Fructose
GA3P Glyceraldehyde 3-phosphate
1,3 BPGA 1,3-Bisphosphoglycerate
3PGA 3-phosphoglycerate
Pyr Pyruvate
Lac Lactate
6PGL 6 phosphoglycerate
R5P Ribose 5 phosphate
Rib Ribose
Hpx Hypoxanthine
UAC Uric acid
P-pyr Phosphopyruvate
Oaa Oxaloacetate
Cit Citrate
Akg α-ketoglutarate
Succ Succinate
AcCoA AcetylCoA
CT Cytrulline
Orn Ornithine
ArgSucc ArgininoSuccinate
Cr Creatinine
CP Carbamoyl phosphate
TCA Tricarboxylic Acid Cycle
LC3 Microtubule-associated protein 1A/1B-light chain 3
FDG Fluorodeoxyglucose
FLT Fluorothymidine
PET Positron Emission Tomography
Q-TOF Quadrupole-Time of flight
GC/MS gas chromatography-mass spectrometry
GLS Glutaminase
PCK2 Phosphoenolpyruvate carboxykinase 2
PC Pyruvate carboxylase
TK1 Thymidine Kinase 1
HK Hexokinase
LDH Lactate dehydrogenase
AMPK Adenosine monophosphate kinase
ROS Reactive oxygen species
ATP Adenosine 5'-triphosphate
GMP Guanosine monophosphate
CQ Chloroquine
BPTES Bis-2-[5-phenylacetamido-1,2,4-thiadiazol-2-yl]ethyl sulphide
MSTFA (N-Methyl-N-(trimethylsilyl) trifluoroacetamid
Aden Adenosine
Ade Adenine
Xan Xanthine
Spd Spermidine
Gua Guanosine
Putr Putresceine
OXPHOS Oxidative phosphorylation
Ala Alanine
Arg Arginine
Asn Asparagine
Asp Aspartate
Cys Cysteine
Gln Glutamine
Glu Glutamate
Gly Glycine
Ile Isoleucine
Leu Leucine
Lys Lysine
Met Methionine
Phe Phenylalanine
Pro Proline
Ser Serine
Thr Threonine
Trp Triptophane
Tyr Tyrosine
Val Valine
NEAA Non-essential amino acid

Metabolite extraction

For metabolite extraction from NIH3T3 and NIH3T3 K-Ras, cell lines were plated at 3000 cells/cm^2 in 6-well plates with normal growth medium. After 54 or 144 hrs, cells were quickly rinsed with NaCl 0.9% and quenched with 0.4 ml ice-cold methanol. An equal volume of water was added, and cells were collected by scraping with a pipette tip. Cells were sonicated 5 seconds for 5 pulses at 70% power three times. One volume of chloroform was added, and cells were vortexed at 4°C for 20 min. Samples were centrifuged at 12000 *g* for 10 min, and the aqueous phase was collected in a new tube and evaporated under airflow at 37°C.

Derivatization

Dried polar metabolites were dissolved in 60μl of 2% methoxyamine hydrochloride in pyridine (Pierce) and held at 40°C for 6 h. After dissolution and reaction, 90μl of MSTFA (N-Methyl-N-(trimethylsilyl) trifluoroacetamid) was added and samples were incubated at 60°C for 1h.

For cell culture, GC/MS analysis was performed using 6890 GC system combined with 5975BMS system (Agilent Technologies) equipped with a 30-m DB-5MS capillary column operating under electron impact (EI) ionization at 70eV. 1μl of sample was injected in splitless mode at 250°C, using helium as the carrier gas at a flow rate of 1 ml/min. The GC oven temperature was held at 70°C for 2 min and increased to 325°C at 10°C/min.

Immunofluorescence microscopy

Normal and Transformed fibroblasts were analyzed by fluorescence microscopy using Cyto-ID® Autophagy detection kit (Enzo Life Sciences) according to the manufacturer's protocol, and LAMP1. For LAMP1 staining, cells were fixed for 10 min in 4% paraformaldehyde (PFA) in PBS 1X. Next they were permeabilized for 30 min in 0.05% (w/v) saponin, 0.5% (w/v) BSA and 50 mM NH4Cl in PBS (blocking buffer). The cells were incubated for 1h with the primary antibody LAMP1 (1:500) (Abcam), washed three times in PBS 1X, incubated for 1h with the secondary antibody (Alexa-labelled), washed 3 times with PBS 1X, stained with Hoechst (0.1μg/ml) for 2 min, washed three times in PBS 1X and finally mounted with DABCO (Sigma Aldrich).

Western blotting analyses

Cells were lysed in a buffer containing 150 mM NaCl, 0.5% NP-40, 1% glycerol, 50 mM HEPES (pH 7.5), 5 mM ethyleneglycol tetracetate (EGTA), 1mM phenylmethylsulphonyl (PMSF) 50 mM NaF and a cocktail of protease inhibitors (Roche) and incubated for 30 min on ice. Extracts were centrifuged at 12000 *g* for 20 min. Protein concentration of supernatant was measured using the 2-D Quant Kit (GE Healthcare) following the manufacture's procedure, using bovine serum albumin (contained in the kit) as a standard. 30 μg of proteins from the total cellular extract were subjected to electrophoresis in sodium dodecyl-sulfate (SDS) polyacrylamide gels. Proteins were then transferred to nitrocellulose membrane (GE Healthcare) by electroblotting and incubated with antibodies against AMPK, phospho-AMPK, ULK, phospho-ULK, LKB1 (all from Cell Signaling Technology) and Vinculin (Pierce), and then incubated with peroxidase-coupled secondary antibody (LiStar Fish). After washing, the reaction was visualized with ECL (GE Healthcare) followed by exposure to a X-ray film.

Electron microscopy analysis

For electron microscopy, cells were grown in 35mm dishes in complete growth medium. After 54h under normal growth conditions (4mM Gln) or nutrient deprivation (0.5mM Gln) cells were fixed with 2% glutaraldhyde buffered with 0.1M sodium cacodylate at pH 7.4 for 2h at room temperature. Fixed cells were detached using cell scraper, collected into microtubes and centrifuged. Cell pellets were washed three times in 0.1M sodium cacodylate at pH 7.4, post-fixed in 1% OsO4 at room temperature in the same buffer, washed three times in distilled water and stained with 0.5% uranyl acetate o.n. at 4°C. Pellets were dehydrated in graded steps of ethanol (50%, 70%, 90%, 100%), two times with 100% acetone and embedded into Epon. Section (60nm thick) were cut on a Leica UC7 ultramicrotome and examined with Fei Tecnai 12 BioTwin Spirit transmission electron microscope.

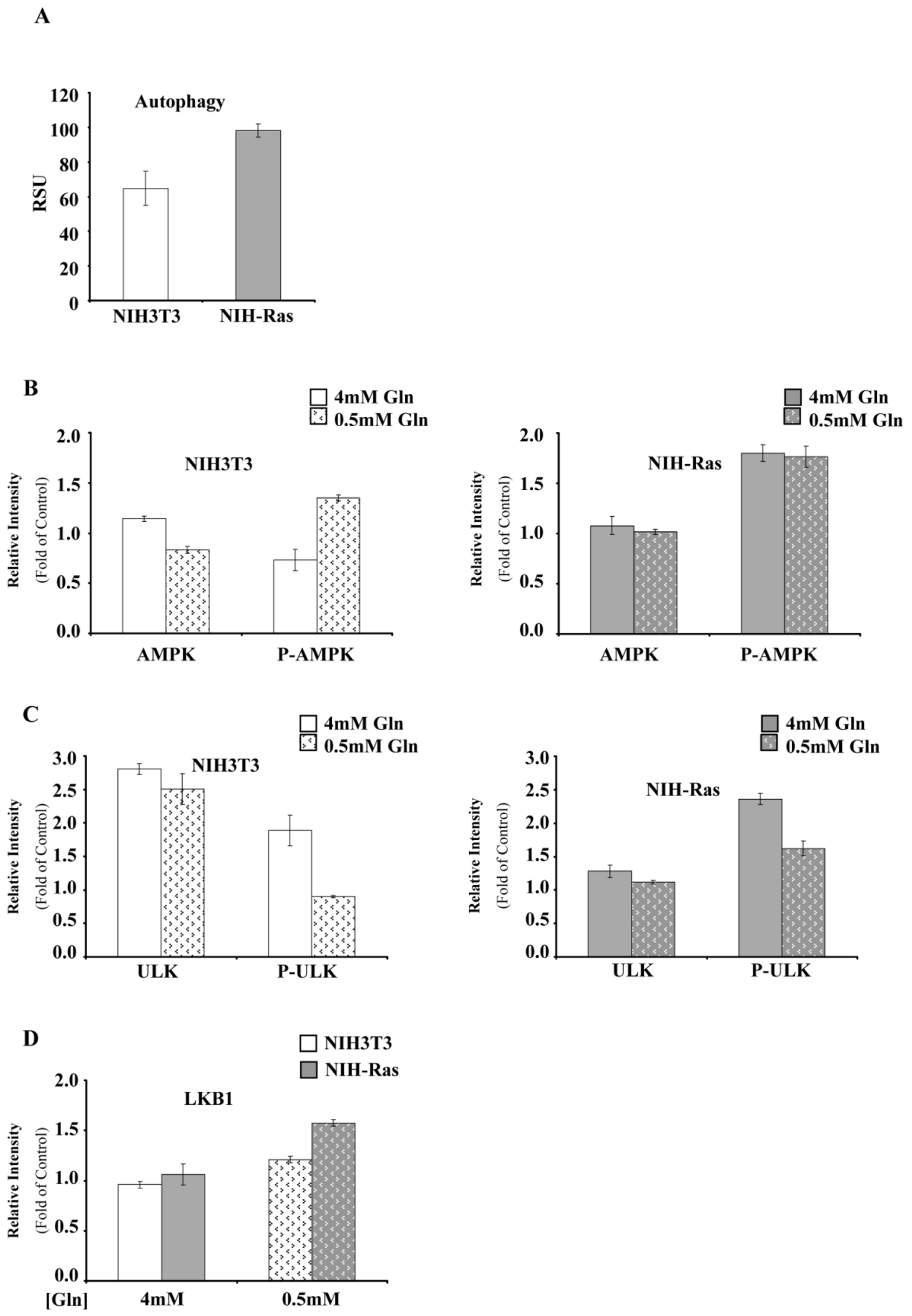

Supplementary Figure S1: A. Intensity of basal autophagy fluorescence in NIH3T3 (□) and NIH-Ras (■) cells grown in 4mM glutamine for 54h, measured by Cyto-ID® Autophagy detection kit. Error bars indicate SD (n=3). **B-C** and **D.** Relative quantitative analysis of protein levels after Western blot film acquisition by scanner and densitometric analysis using Image J program. The densitometry values obtained for each protein were normalized by using the values of the corresponding vinculin or actin. Results are the mean of at least triplicate determinations with SD indicated.

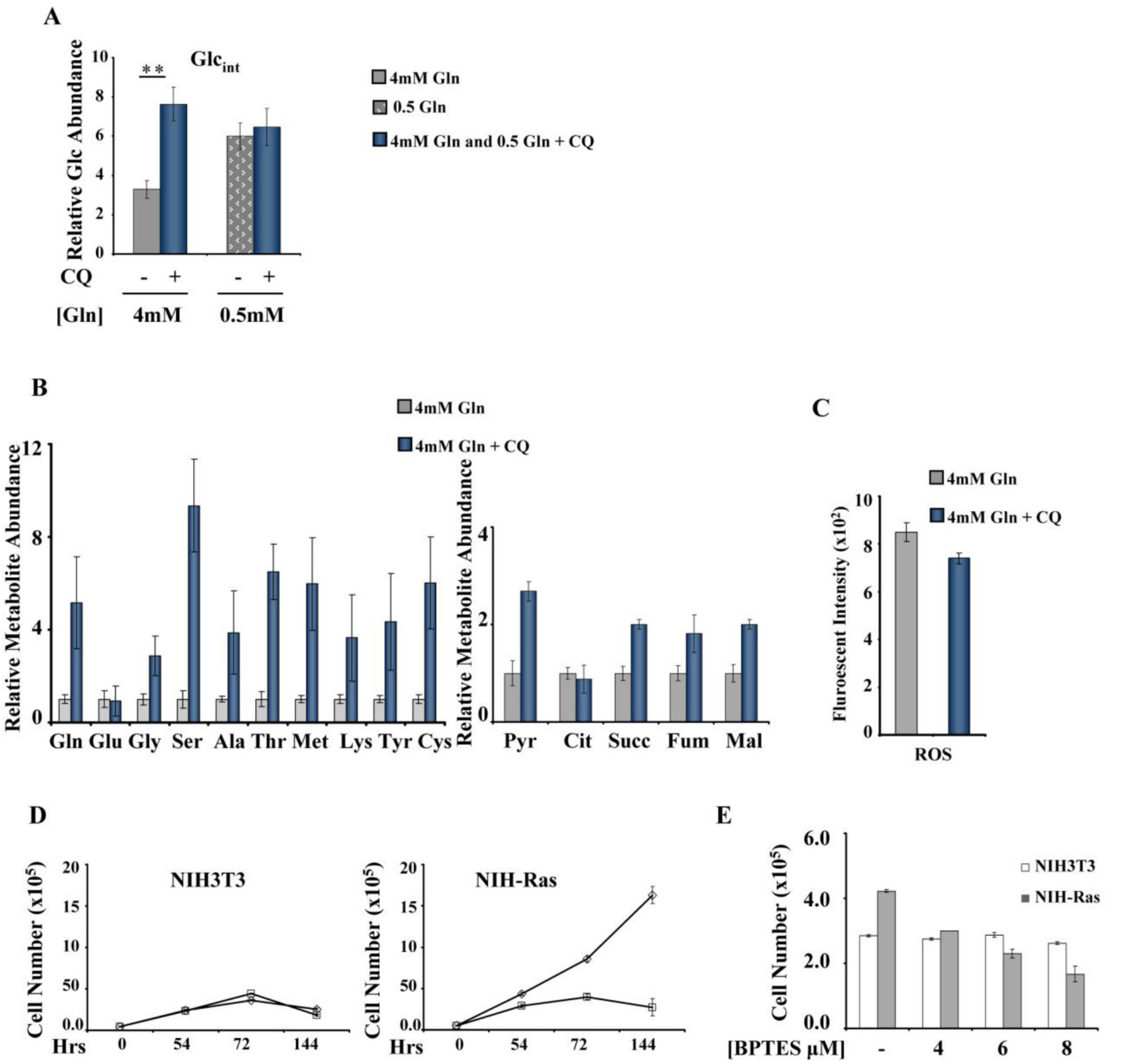

Supplementary Figure S2: A. Relative glucose abundance measured by GC/MS in NIH-Ras cultured with or without Gln for 54h. Error bars indicate SD (n=5). P-value were calculated using the Student's *t*-test (P **< 0.01). **B.** Relative metabolites abundance measured by GC/MS in NIH-Ras cultured in 4mM Gln or 4mM Gln plus CQ for 54h. Error bars indicate SD (n=5). **C.** Intracellular ROS levels measured by using 5mM DCFH2-DA staining of NIH-Ras grown in medium containing 4mM Gln or 4mM Gln plus CQ for 54h. Error bars indicate SD (n=3). **D.** Proliferation curve of NIH3T3 (left panel) and NIH-Ras (right panel) mouse fibroblasts. Cells were plated at 3000 cells/cm^2 in 6-well plates in normal medium. Culture medium was replaced after 18h with normal medium containing 4mM Gln (◊) or 0.5mM Gln (□-low Gln). Cells were collected and counted at indicated time points. **E.** Effects of Gln inhibitor BPTES (■) in NIH3T3 and NIH-Ras mouse fibroblasts. Cells were plated at 3000/cm^2. After 18h cells were treated with different BPTES concentrations (4-6-8µM) and counted at 54h after treatment. Error bars indicates SD (n=3).

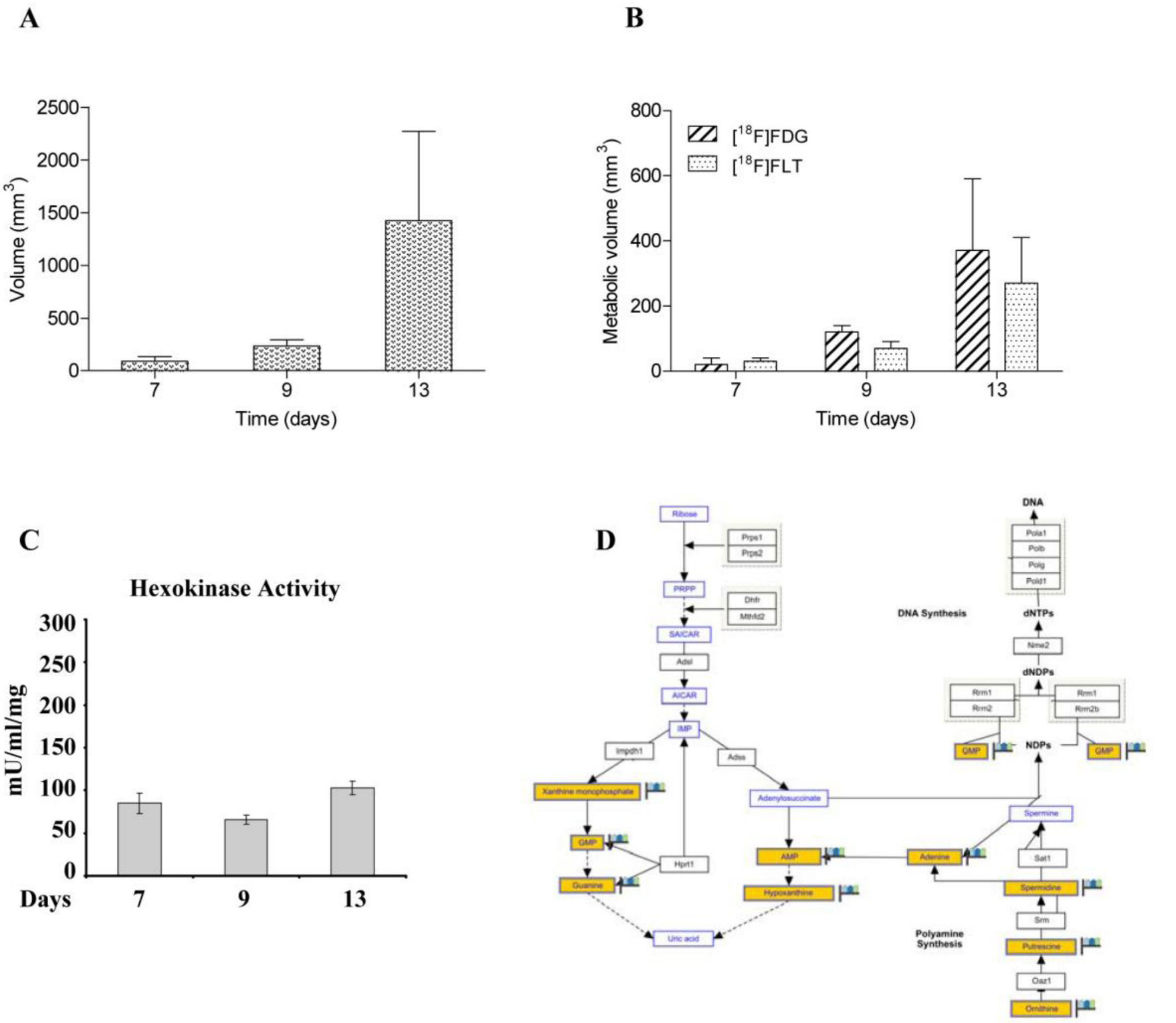

Supplementary Figure S3: A. Analysis of changes in tumor size measured at caliper after 7, 9 and 13 days s.c. injection of the NIH-Ras cancer cell fibroblasts. **B.** [^{18}F]FDG and [^{18}F]FLT metabolic volume measured using PET imaging at 7, 9 and 13 days after s.c. injection of the NIH-Ras cancer cell fibroblasts. Error bars indicate SD (n=5). **C.** Hexokinase enzyme activity of K-ras transformed cells grown in xenografts nude mice for 7, 9 and 13 days. HK enzyme activity was measured by enzymatic assay and normalized to protein content. Error bars indicate s.e.m. (n=3). **D.** Schematic representation of nucleotides metabolism. Nucleotides metabolic map was from KEGG, visualized in Pathway Architect, a Mass Profiler Professional software tool. Metabolites marked in yellow indicate matches with the input entity list.

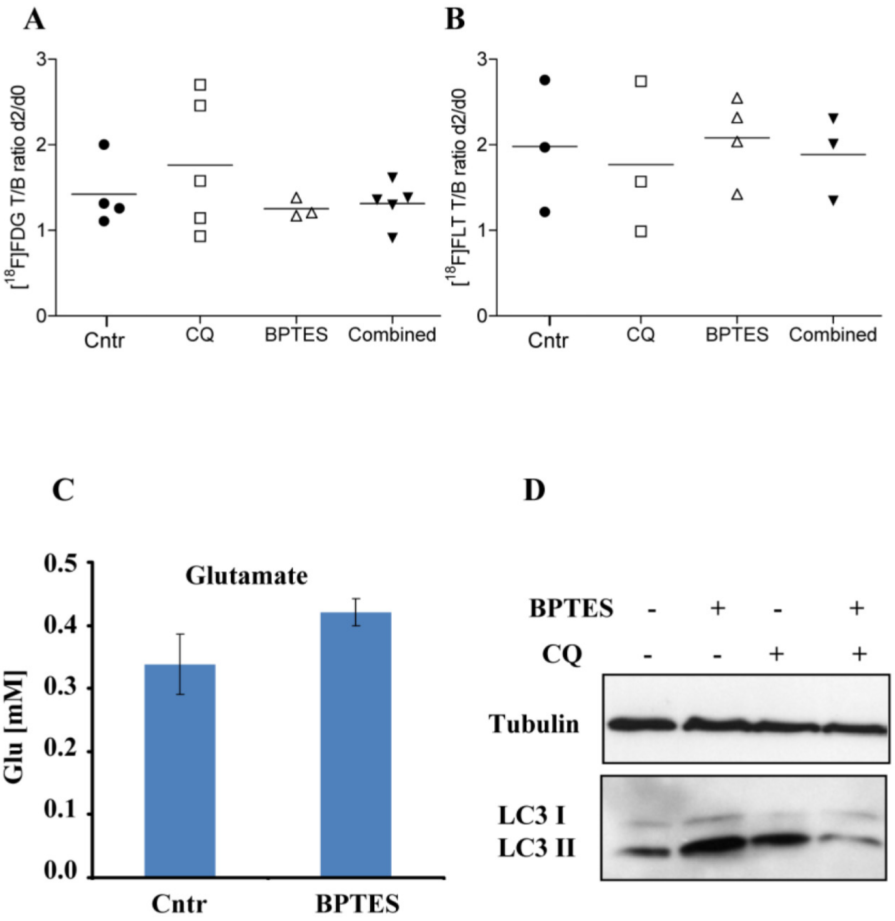

Supplementary Figure S4: **A** and **B.** PET quantification after treatment. Quantitative analysis of changes in [^{18}F]FDG (left) and in [^{18}F]FLT (right) T/B ratio. Values are calculated as ratio between T/B pre and 48h post treatment in vehicle (Cntr), and treated groups (CQ, BPTES and Combined). For each group, 3-5 animals performed [^{18}F]FDG or [^{18}F]FLT acquisition. **C** and **D.** Glutamate metabolite concentration of K-ras transformed cells growth in xenografts nude mice for 7 days and 48h post treatment. Glutamate was measured by enzymatic assay and was normalized to protein content. Error bars indicate SD (n=3). Protein expression analysis of NIH-Ras tumors after single or simultaneous treatments of BPTES and CQ. Western blotting performed using LC3 I and II and tubulin antibodies.

A

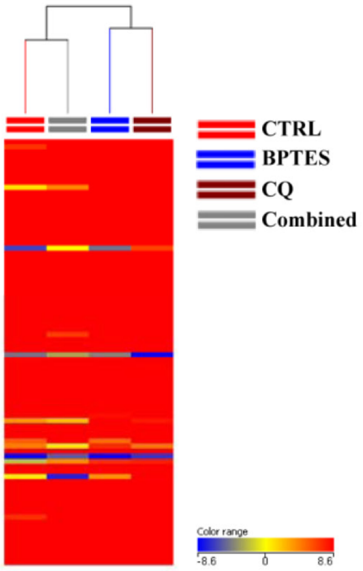

B

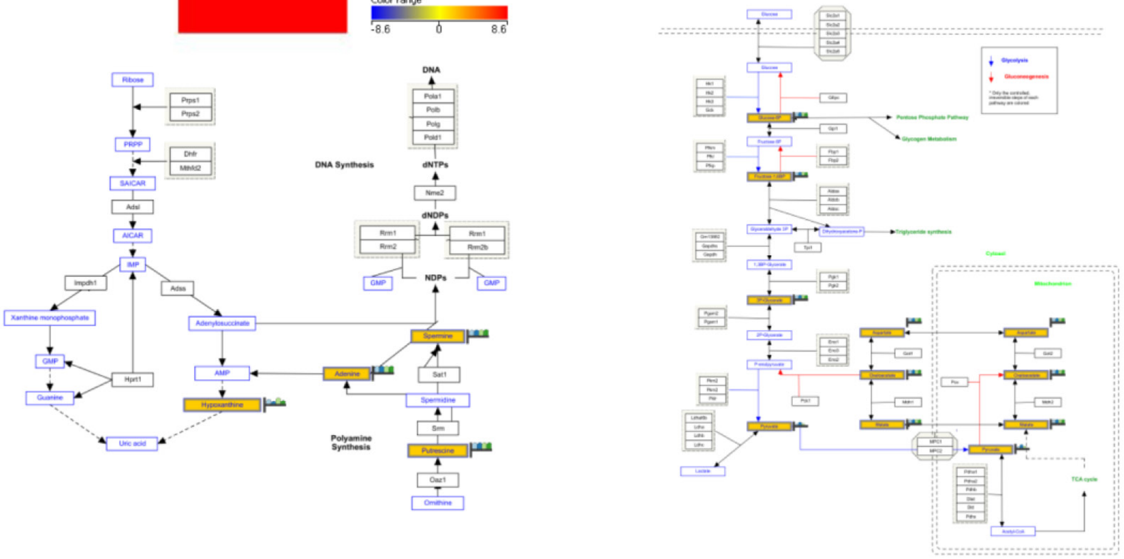

C

COMPOUND	*CTRL*	*BPTES*
Cr	1,256869778	17,32942738
β-Ala	12,62575622	13,87544888
Asp	20,08723489	20,76885838
Urea	15,09562372	16,26510050
Fru	17,40336589	18,05133638
Glc	16,80212289	18,64300225

D

COMPOUND	*CTRL*	*CQ*
Urea	15,09562372	16,24206250
Glc	16,80212289	18,55390856
Uric	9,767568478	11,28507722
β-Ala	12,62575622	13,87074656
Hyp	11,92062389	15,34794128

Supplementary Figure S5: A. Comparison of samples subjected by statistical analysis using Mass Profiler Professional (MPP) software. The dendrogram was produced by applying a hierarchical clustering algorithm. **B.** Schematic representation of nucleotides, glycolysis and TCA cycle metabolism. Metabolomic maps were from KEGG, visualized in Pathway Architect, a Mass Profiler Professional software tool. Metabolites marked in yellow indicate matches with the input entity list. **C** and **D.** The tables show quantitative values of relative metabolites abundance obtained by unpaired t-test analysis using Mass Profiler Professional.
